# Trace Element Concentration and Stable Isotope Ratio Analysis in Blueberries and Bilberries: A Tool for Quality and Authenticity Control

**DOI:** 10.3390/foods10030567

**Published:** 2021-03-09

**Authors:** Linards Klavins, Inessa Maaga, Maris Bertins, Anne Linn Hykkerud, Katja Karppinen, Česlovas Bobinas, Heikki M. Salo, Nga Nguyen, Henriikka Salminen, Karina Stankevica, Maris Klavins

**Affiliations:** 1 Department of Environmental Science, University of Latvia, LV-1004 Riga, Latvia; linards.klavins@lu.lv (L.K.); inessa.maaga@lu.lv (I.M.); karina.stankevica@lu.lv (K.S.); 2 Faculty of Chemistry, University of Latvia, LV-1004 Riga, Latvia; maris.bertins@lu.lv; 3 Norwegian Institute of Bioeconomy Research, 1433 Ås, Norway; Anne.Linn.Hykkerud@nibio.no; 4 Department of Arctic and Marine Biology, UiT The Arctic University of Norway, 9037 Tromsø, Norway; katja.h.karppinen@uit.no; 5 Lithuanian Research Centre for Agriculture and Forestry, 58344 Kėdainiai, Lithuania; ceslovas.bobinas@lammc.lt; 6 Ecology and Genetics Research Unit, University of Oulu, 90570 Oulu, Finland; Heikki.M.Salo@oulu.fi (H.M.S.); Thi.Nguyen@oulu.fi (N.N.); Henriikka.Salminen@oulu.fi (H.S.)

**Keywords:** blueberries, trace elements, heavy metals, light stable isotope ratio, pollution, authenticity, bilberries

## Abstract

*Vaccinium* genus berries—wild bilberries (*Vaccinium myrtillus* L.) and cultivated highbush blueberries (*Vaccinium corymbosum* L.)—are consumed worldwide, and their consumption has a trend of stable increase. Thus, considering their wide use in ethnomedicine, for juice and jam production, as functional food, as well as their use in preparations of extracts which have application potential in pharmaceutical and cosmetics industries, studies regarding the composition of these berries are of special importance. The aim of this study is to characterise the elemental and isotopic composition, as well as variation in element concentration in bilberries gathered from different sites in Northern Europe and in commercially available blueberry samples from across the World. Furthermore, our aim was to develop tools for authenticity and quality control of these berries. The elemental composition of berries was analysed using inductively coupled plasma with optical emission detection (ICP-OED), while isotope ratio mass spectrometry (IRMS) was used for the determination of isotope ratio values. The results demonstrated detectable differences between macro- and microelement values in bilberries. IRMS analysis of blueberries revealed significant differences in isotope ratios based on the place of origin, indicating the possibility to use this analytical method for authenticity testing. In none of the samples, pollution was detected, even though there were indications of different growth conditions and geochemical differences affecting bilberry composition.

## 1. Introduction

Consumers are becoming increasingly health-conscious, and as a consequence, health-beneficial foods are gaining more and more attention as part of the human diet. This has led to a worldwide increase in the consumption of berries because, besides having an attractive appearance and a balanced sweet-sour taste, they present an important dietary source for essential vitamins, minerals, dietary fibre and bioactive compounds, such as phenolic acids, flavonoids and tannins, which have numerous health-beneficial properties [1]. The growing popularity of berry-rich diet has led to an increase in the production and consumption of two *Vaccinium* genus berries, namely wild bilberries (*Vaccinium myrtillus* L.) and cultivated blueberries (*Vaccinium corymbosum* L.). For instance, in 2019, the commercial production of blueberries reached 676,313 tons in Americas, and 136,495 tons in Europe [2]. Additionally, the gathering of wild bilberries in Northern Europe and Russia reaches several hundreds of tons yearly with a stably increasing trend. The various health-promoting properties of blueberries and bilberries are widely recognised since their consumption can reduce the risk of many infectious and degenerative disease [3,4]. For instance, blueberry and bilberry fruits have been used as fresh, dried and as juice for the alleviation of indications related to the gastrointestinal tract and diabetes, and herbal supplements containing these berries are available in the market as aids to improve vision and to treat diarrhoea because they have antimicrobial, anti-inflammatory and antioxidant properties [5,6,7,8]. The biological and pharmacological activity of blueberries and bilberries are associated with their high content of polyphenolic compounds, especially anthocyanins, and their capacity to reduce oxidative stress by scavenging free radicals [9]. Additionally, berry lipids, such as terpenes, sterols, unsaturated fatty acids and waxes, contribute to the biological activities of these berries and their extracts [10].

Another group of valuable components in blueberries and bilberries are the elements they contain (including K, Ca, Mg, P, Fe, Mn and Zn). These are essential for various functions in the human body and are therefore essential components of the human diet as well. At the same time, many non-essential inorganic elements (such as Cd, Pb and As) might be indicative of the presence of anthropogenic pollution [11]. For these reasons, studies regarding the element composition of berries, as well as analyses which focus on the presence of toxic elements, are of a special importance. The adverse effects of environmental pollution sources, such as metal processing industries, on the presence of toxic trace elements in berries have been studied. For instance, significantly elevated concentrations of trace elements in comparison with background pollution were found in berries sampled in the vicinity of ferrochrome and stainless-steel factories in Northern Finland [12,13]. Also, as a consequence of mining and metal processing industries, high concentrations of Ag, As, Be, Bi, Br, Cd, Hg, I, Ni, Pb, Sb and Tl have been found in berries growing at mining areas in Northern Sweden [14]. Recently, the impact of wood ash applications on the elemental composition of berries was studied and risks related to the increasing concentrations of trace elements were found [15]. Another aspect of the berry quality studies is the evaluation of element concentrations in berry-containing products available on the market [16]. However, in the case of products, it is nearly impossible to relate the found concentrations with the origin of the samples because the products may be subject to adulteration. Thus, there is a need to further develop the methods utilised for berry origin authentication.

As the growth conditions, as well as metal accumulation patterns for different plant species vary, it is important to study contamination levels in species which are of importance for human consumption, such as blueberries and bilberries. Only a few studies with a limited number of elements studied are dedicated to the elemental composition of trace elements in blueberries and bilberries [15,17,18]. Another important aspect in the study of elements is related to the possibility to identify the origin and cultivation practices of berries, based on compositional analysis. The aim of this study was to characterise the elemental and isotopic composition and concentration variability of elements, as well as develop tools for the authenticity and quality control of bilberries gathered in Northern Europe and commercially available blueberry samples from across the world.

## 2. Materials and Methods

### 2.1. Sampling

The fruits of wild bilberries were collected during 2019–2020 vegetation seasons from 26 sampling sites in the territory of Latvia, and from a total of nine sampling sites in Norway, Finland and Lithuania, three in each country. Samples of different varieties of highbush blueberries were sampled in 2018 from a commercial blueberry farm ‘Strelnieki’ (Latvia). After collection, the samples were frozen, vacuum packaged and stored in a freezer at −20 °C for a maximum of three months. Commercial samples of fresh blueberries were obtained from supermarkets during 2018–2020. The country of origin was indicated on the labels and the sample set included blueberries from Peru, Argentina, Uruguay, Chile, Morocco, Spain, Germany, Poland and Latvia. After purchase, the blueberries were frozen and stored in a freezer at −20 °C. For analysis, the samples were lyophilised and homogenised using a pestle and mortar. Agate pestle and mortar were used for homogenising the samples for isotope ratio mass spectrometry (IRMS) analysis.

### 2.2. Analysis of Trace Elements

Dried berry samples (1.00 g) were weighed into Teflon tubes followed by the addition of 8 mL 65% HNO_3_ (Sigma Aldrich, Darmstadt, Germany) and 2 mL 30% H_2_O_2_ (Enola, Riga, Latvia). The tubes were closed (to provide high pressure) prior to sample digestion using the Ethos Easy microwave system (Milestone, Sorisole, Italy) at 200 °C for 30 min. The resulting samples were diluted to 50 mL with deionised water (7.4 µS/cm) (Millipore, Burlington, MA, USA). The concentrations of inorganic elements were determined by iCAP 700 series inductively coupled plasma spectrometer with optical emission detection (ICP-OED) (Thermo Scientific, Waltham, MA, USA). The elements determined were Al, As, B, Ba, Ca, Co, Cr, Cu, Fe, K, Li, Mg, Mn, Mo, Na, Ni, Pb, Rb, Sb, Se, Sn, Sr, Ti, Tl, V and Zn. Detection limit was 1–10 ppb for Al, Ca and Fe, 0.1–1 ppb for Mg, K and Na, and 0.1 ppb for all other elements. Concentrations were expressed per dry weight of berries. The accuracy of the analysis of berry samples was verified by the following certified reference materials: SRM 3287—Blueberry (fruit) (National Institute of Standards and Technology (NIST, Gaithersburg, MD, USA) and the National Institutes of Health Office of Dietary Supplements (NIH ODS, Bethesda, MD, USA)). The difference between the data of berry samples analysed and the reference materials was generally lower than 15% for all elements.

### 2.3. Light Stable Isotope Ratio Analysis (δ^13^C, δ^15^N, δ^18^O)

For the determination of the stable isotope ratios, dried berry samples (5.0 mg) were weighed into tin capsules for C and N analyses, and 1.0 mg into silver capsules for O analysis (EuroVector, Pavia, Italy). After weighing, the capsules were carefully folded. All samples were prepared in triplicate. Glutamic acid (C and N analysis) and sucrose (O analysis) laboratory standards (Sigma Aldrich, Darmstadt, Germany) were used for calibration (0.2, 0.5, 0.8, 1.0 and 1.5 mg). To monitor the stability of the obtained values, one glutamic acid or sucrose control sample (1.0 mg) was analysed after every 10 samples. To verify trueness of the obtained results, glutamic acid (USGS-40, d^13^C —26.39 ± 0.04‰ VPDB, d^15^N —4.52 ± 0.06‰ AIR, *w*C = 40.8%, *w*N = 9.52%) and benzoic acid (IAEA-601, d^18^O —23.14 ± 0.19‰ VSMOW, *w*O = 26.2%) reference materials were used. The ratio of C, N and O isotopes in samples was measured on an isotope ratio mass spectrometer Nu Horizon (Nu Instruments, Wrexham, United Kingdom), acceleration voltage: 5 kV, mass range: 2–100 Da, mass dispersion: >30 cm) using the Euro EA3000 element analyser (EuroVector, Pavia, Italy) with quartz combustion column filled with chromium (III) oxide and silvered cobaltous oxide (1030 °C) and a quartz reduction tube filled with copper shards (650 °C) for the determination of C and N isotope ratio. For the determination of the O isotope ratio, a high-temperature element analyser unit, HTEA PyrOH (EuroVector, Pavia, Italy), was used with outer ceramic tube and inner glassy carbon tube filled with glassy carbon chips and nickelled carbon (1420 °C). The results were processed by the Nu Stable Control Software v1.69 (Nu Instruments).

### 2.4. Statistical Analysis of Results

The Kruskal–Wallis nonparametric test was used to detect differences among element concentrations in samples with different origin or variety. Statistical data analysis, including principal component analysis (PCA) of metal concentration and stable isotope ratios, were done using statistical data discovery software SAS JMP^®^, version 14 (SAS Institute Inc., Cary, NC, USA).

## 3. Results and Discussion

In blueberry and bilberry wet digested samples, total element concentrations were determined by the ICP-OED. Since highbush blueberries are cultivated, many varieties that differ in berry ripening time, size, taste and other properties are available [19]. Differences in element concentrations in eight varieties of blueberries growing on peat soil in one location (biological farm in Latvia) were compared (Table 1). Our results show that blueberries are a rich source of mineral elements, especially K, Ca, Mg, P and S. Blueberries as a source for K, Ca, Mg and Mn has been shown in other studies as well [17,18]. However, the concentrations of toxic trace elements (Co, Se, Pb, Ni, Cr, Co, As) has been shown to be lower in berries growing on soils with elevated metal concentrations [20]. For nearly all elements, differing concentrations in berries were found in the studied cultivars. This reflects the impact of the type of cultivar on the mineral composition of blueberries.

As blueberries are cultivated worldwide, it was of interest to compare their elemental composition in commercially available samples as well. Elemental composition was analysed in samples obtained from nine countries during the period 2018–2020 with 24 elements analysed altogether (Table 2). In all studied samples, the highest concentrations were found for the elements important for human consumption, namely K, Ca, Mg, Fe, P, S and Mn. However, their variability was high, and for example, the concentration of K in berries from South America or North Africa was twice as high compared to samples from North Europe where cultivation usually takes place in mineral poor peat soils. In commercially available cultivated berry samples, values of trace elements were found to be low, but comparable with element concentration values found in other studies [17,18]. However, variability in some of the trace elements in some of the berry samples was significantly higher than in others. For example, in samples from Europe, the concentration of V was 0.03 ± 0.01, whereas its concentration was nearly five times higher in samples from outside the Europe (Table 2). Similar differences in concentrations were found for other trace elements as well, such as Mo, Se, Pb and Ni. Considering the amounts of blueberries that are cultivated and consumed, and the toxicity values of the studied elements [21], it can be concluded that the trace element concentrations found in the commercially available berry samples do not pose a risk to human health. However, considering the high volumes of blueberries sold on the markets worldwide, it would be important to establish quality criteria for the composition of trace elements in them.

**Figure 1:**
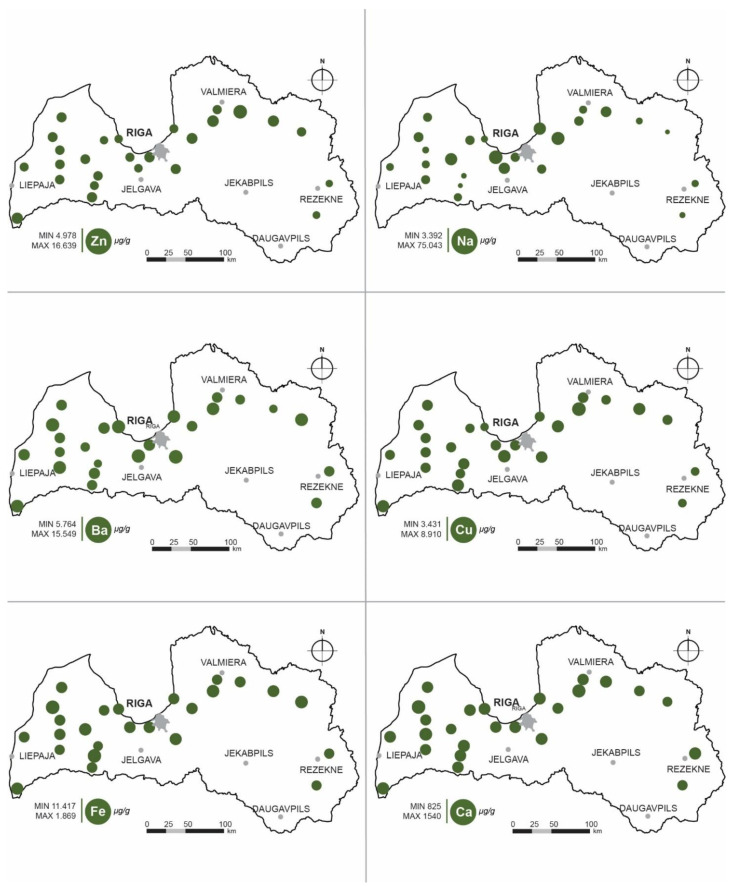
Elemental composition of bilberries in Latvia.

The values of element concentrations in bilberries from the territory of Latvia (64,000 km^2^) also demonstrate variability (Figure 1). Maximal values of the studied elements are associated with the known local and regional environmental pollution sites. For example, elevated concentrations in the western part of Latvia indicate the presence of industrial pollution from the long-time functioning cement production, as well as metallurgical factories, as has been shown previously [22,23,24]. Another major factor influencing bilberry elemental composition are the geochemical differences in soils of Latvia, for example differences in Ca/Mg ratio in different parts of the country [25]. As has been stated in earlier studies, the geochemical characteristics of soil are one of the major factors affecting element composition of bilberries [13,16,26]. Nevertheless, the variability of concentrations of trace elements in wild bilberries reflect a specific pattern of metal and other trace element concentrations in soils and can thus be considered as specific for the territory of Latvia. At the same time, the concentrations of toxic elements and heavy metals found in our study in all locations can be considered low, especially when compared to the concentrations found in previous studies [7,14,18,26]. Therefore, bilberries grown in Latvia can be considered as a valuable source of mineral substances and essential elements.

Comparison of the elemental composition of bilberries sampled from the selected natural forest stands in the Baltic Sea region countries (Latvia, Lithuania and Finland) and Norway demonstrates larger variability than the nationwide sampling in Latvia (Table 3). Among the studied berries from the different countries, major elements Na, Mg, P, S, B and Ba, as well as trace elements Cr and Se, were found in the highest concentrations in Norway, whereas Ni and Co were found in the lowest concentrations (Table 3). Berries gathered in Finland had the lowest concentration of S, Fe, Zn and As, while the concentration of Co showed values twice as high compared to berries from other countries. Berries sampled from Lithuania had twice as high concentrations of V and Pb than the other countries, indicating possible anthropogenic pollution near the sampling sites. The obtained results indicate geomorphological specificities of element composition in berries from different regions in the Baltic Sea region and Norway.

**Figure 2:**
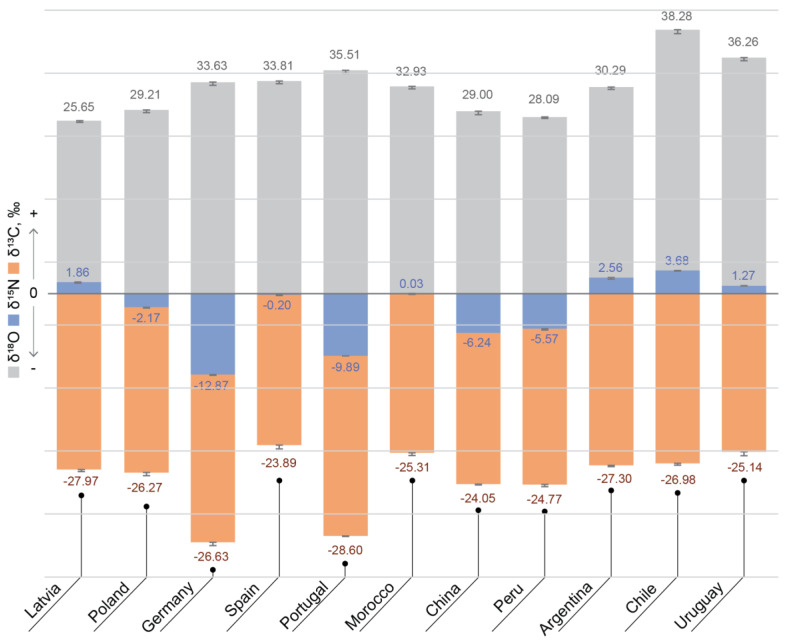
Light stable element isotope (δ^13^C, δ^15^N, δ^18^O) ratio values in blueberries from different countries.

**Figure 3:**
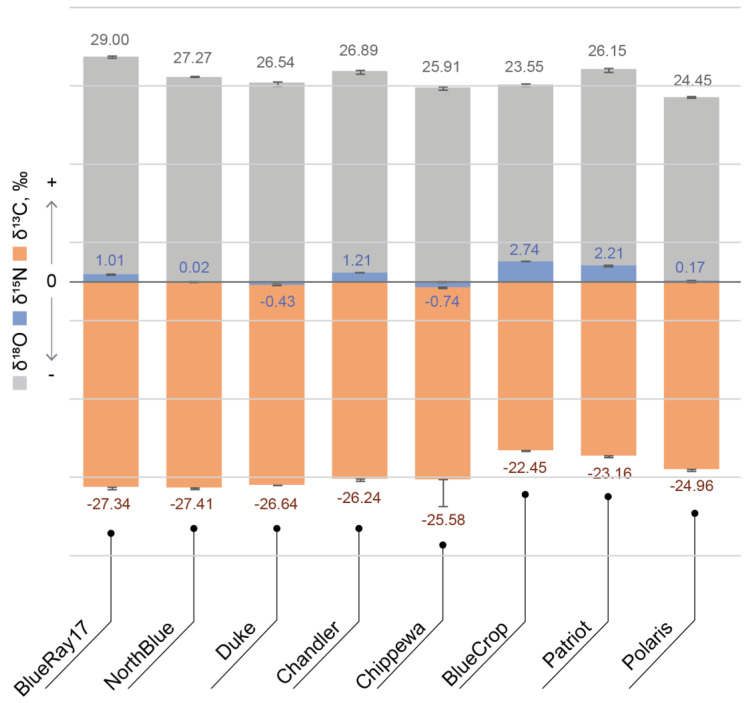
Light stable element isotope (δ^13^C, δ^15^N, δ^18^O) ratio values in different varieties of blueberry from one location in Latvia. Error bars represent standard error between the replicates (*n* = 5).

Considering the significant effect of origin on the composition of blueberries and bilberries, it is of importance to develop authentication methods for berry origins and the detection of potential adulteration. The use of elemental composition analyses, as well as light stable element isotope (δ^13^C, δ^15^N, δ^18^O) ratio analyses, which have been previously used for the successful determination of authenticity and origin of wine and table grapes [27], goji berries [28] and other berries [29], could be suggested as a prospective approach for blueberries and bilberries as well. Figure 2 shows the light stable element isotope (δ^13^C, δ^15^N, δ^18^O) ratio values in commercial blueberry samples from different countries. The most significant changes appear in the δ^15^N values (−12.9–3.7‰). Lesser, but nevertheless significant changes can be seen in the δ^18^O values (25.7–38.3‰), whereas the lowest changes are in the δ^13^C values (from −23.9 to −28.6‰). δ^15^N is most commonly used as a nitrogen cycle indicator in different plants [30] and its values are dependent on many factors, such as the availability of nitrogen in soil, the nitrogen source in plants, atmosphere, soil, fertiliser and nitrogen uptake processes in plant itself [31]. However, in commercially grown plants, δ^15^N is an indicator of fertilization practices. Its values are not characteristic for large geographical areas but are more specific to each location (farm). The comparison of δ^15^N values in commercially available blueberries in this study (Figure 2) demonstrates differing patterns of blueberry fertilization. In some samples, the values vary from −12.87 to 3.68 ‰, whereas in the case of organic farming with minimal fertiliser application, the values range from −0.74 to 2.74‰. The latter presents the case of different blueberry cultivars sampled from a specific farm in Latvia (Figure 3). Also, in wild bilberries from different locations in Latvia (sampled from forests), δ^15^N values range from −2.20 to 5.21‰. Thus, this interval of δ^15^N values can be considered as indicative for natural berry cultivation (growth) conditions.

**Figure 4:**
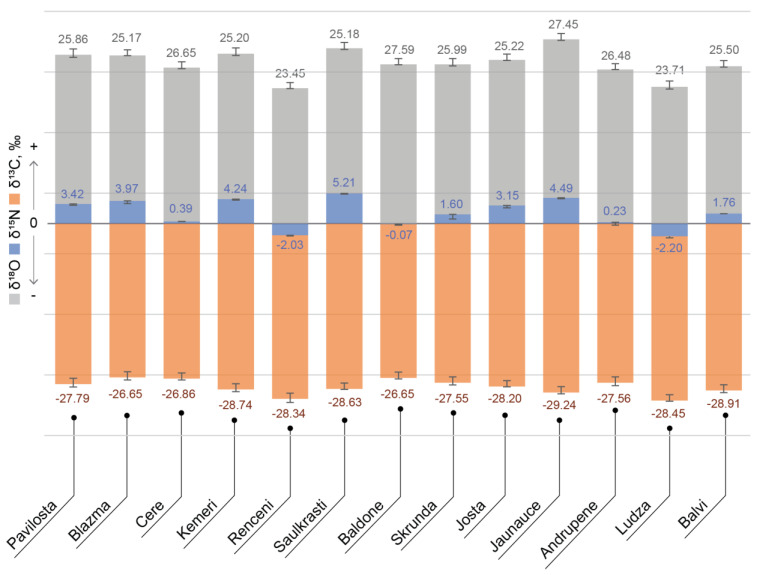
Light stable element isotope (δ^13^C, δ^15^N, δ^18^O) ratio values in bilberries from different locations in Latvia. Error bars represent standard error between the replicates (*n* = 5).

δ^18^O values are more characteristic to the geographical location and are usually associated with the climatic conditions, such as temperature [32]. From the δ^18^O values observed in blueberries from different countries, a pattern based on the δ^18^O water cycle can be seen. Because of natural water cycle and precipitations, the heavy oxygen isotope (^18^O) tends to concentrate more in oceans [33,34], and therefore the lower δ^18^O values indicate a greater distance from the ocean. A similar pattern can be seen in Figure 4; countries with greater distance from the oceans (Latvia, Poland and continental China) have lower δ ^18^O values (25–29‰) compared to the countries closer to oceans and with a warmer climate. The δ ^18^O value of these countries is greater than 30‰.

δ^13^C values are associated with the photosynthetic cycle of plants [35]. However, because all the studied blueberry varieties are C3 plants [29] changes in these values are not very significant. However, there are some observable differences, which are most likely related to the climatic conditions in which the blueberries are growing, such as annual mean temperature and the availability of sunlight.

Based on the results of our study, it is evident that light stable isotope ratio analysis can help identify the origins of the studied berries and provide with a significant amount of information about their growth conditions.

**Figure 5:**
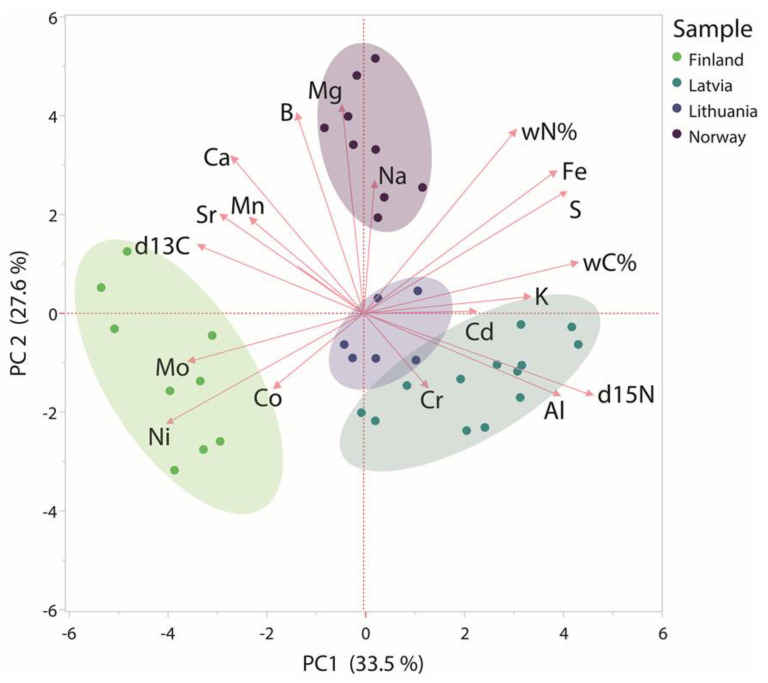
Principal component analysis of metal and light stable isotope contents in bilberries harvested in the Baltic Sea region and Norway (*n* = 9). Ellipses represent 95% confidence intervals.

The data obtained from metal and stable light isotope analysis in bilberries, collected from Finland, Latvia, Lithuania and Norway, were analysed with PCA to visualise possible differences found between the regions of berry harvest. Samples collected from their respective countries clustered together within the matrix, and the analysed data explained 61.1% of the variation (Figure 5). The loadings plot indicates the presence of certain metals at different concentrations in the respective countries, which could be explained by the differences in geochemical composition. The clusters of Lithuania and Latvia showed some overlap (Figure 5) but these neighbouring countries (Baltic States) are known to share closely related types of soils [25]. Nevertheless, despite the slight overlap of Latvia and Lithuania, all the four countries could be clearly separated by the analysis. Therefore, the use of multivariate methods for distinguishing among groups of parameters and their variables could be successfully used to identify specific regions of wild berry origin. However, in order to provide means for the identification of different countries, even more detailed analyses, as well as the establishment of reference databases of isotopic ratios for different regions would be necessary [35]. IRMS data has been used previously to identify the origin of meat [36], extra virgin olive oil [37], as well as fruits and vegetables [38,39]. However, an approach that combines stable isotope ratio analysis with metal analysis could provide with a more specific method for the determination of bilberry and blueberry origins in even greater detail. Furthermore, similarly to previous studies in which IRMS has been used to determine adulteration in fruit juices [40], essential oils [41] and instant coffee [42], such approach could be used for bilberry products, which are often diluted with much cheaper berries, such as cultivated blueberries, elderberries or blackcurrants.

## 4. Conclusions

Element composition and light stable isotope ratios were measured in blueberries of different varieties and different origins, as well as in bilberries collected from the Baltic Sea region (Latvia, Lithuania and Finland) and Norway. Results indicated geomorphological influence on the element composition (major- and minor-elements) of blueberries and bilberries depending on their place of origin. Specific elements, such as Pb, As and Co were found in the berries in varying concentrations and indicate an anthropogenic influence on the growth environment and the presence of possible pollution. Major elements, such as Mn, Fe, Mg and Na in blueberries and bilberries, indicate possible differences in the soil in which the plants have grown and suggest the possibility to use metal analysis as a tool for the determination of berry origins. IRMS analysis provided information on the growth conditions at the site of origin, as well as on the fertilising practices in commercial samples of blueberries. In this study, a total of 24 elements were analysed, which in combination with light stable isotope ratios allowed to differentiate between bilberries grown in countries of the Baltic Sea region and Norway. The demonstrated use of these methods could be utilised as an authenticity testing tool for berry origins.

## Figures and Tables

**Table 1:** Concentrations (mg/kg) of elements in blueberry cultivar samples from Latvia.

Element	Patriot	Polaris	Bluecrop	Northblue	Chandler	Duke	Chippewa	Blueray 17
K ***	4080 ± 136	4823 ± 118	5509 ± 249	5247 ± 190	5626 ± 249	4955 ± 126	6208 ± 94	4706 ± 284
Ca ***	302 ± 27	388 ± 17	386 ± 13	239 ± 53	382 ± 14	432 ± 29	411 ± 37	427 ± 58
Mg ***	265 ± 6	322 ± 14	341 ± 15	286 ± 16	326 ± 8	361 ± 12	291 ± 18	38 ± 22
Na ***	89 ± 5	123 ± 7	57 ± 9	65 ± 9	112 ± 10	208 ± 19	97 ± 14	83 ± 6
P ***	779 ± 22	755 ± 19	713 ± 41	811 ± 31	671 ± 4	658 ± 54	713 ± 27	865 ± 10
S ***	669 ± 15	665 ± 18	601 ± 41	764 ± 29	532 ± 11	546 ± 33	592 ± 26	583 ± 20
Al ***	6.0 ± 1.0	7.3 ± 0.1	7.7 ± 0.5	7.5 ± 0.3	6.2 ± 0.5	5.8 ± 0.4	5.4 ± 0.4	9.2 ± 0.4
B ***	2.17 ± 0.28	3.09 ± 0.25	3.59 ± 0.29	1.41 ± 0.11	2.44 ± 0.17	3.20 ± 0.14	2.12 ± 0.15	2.98 ± 0.29
Ba ***	1.02 ± 0.16	1.09 ± 0.12	1.07 ± 0.06	0.64 ± 0.26	0.75 ± 0.07	1.19 ± 0.32	0.93 ± 0.11	1.46 ± 0.37
Cu ***	2.44 ± 0.29	2.45 ± 0.68	3.51 ± 0.33	1.65 ± 0.20	4.28 ± 0.38	1.73 ± 0.36	2.62 ± 0.61	2.81 ± 0.43
Fe ***	17.4 ± 2.5	20.6 ± 1.6	17.1 ± 0.7	16.1 ± 1.3	20.6 ± 2.0	20.3 ± 2.9	21.7 ± 2.2	18.6 ± 1.4
Mn ***	10.6 ± 0.4	19.1 ± 1.4	15.5 ± 0.9	12.7 ± 1.4	11.0 ± 1.1	12.5 ± 0.5	10.4 ± 0.9	13.5 ± 3.4
Si ***	30.7 ± 5.0	26.1 ± 3.5	41.2 ± 4.1	35.6 ± 2.5	32.8 ± 2.4	23.9 ± 3.4	35.6 ± 4.9	52.2 ± 11.4
Sr ***	0.88 ± 0.15	0.95 ± 0.07	0.87 ± 0.05	0.49 ± 0.11	0.84 ± 0.08	1.37 ± 0.11	0.97 ± 0.07	1.31 ± 0.07
Zn ***	3.7 ± 0.1	3.8 ± 0.3	4.8 ± 0.4	3.9 ± 0.2	3.7 ± 0.1	4.0 ± 0.2	3.7 ± 0.3	4.0 ± 0.01
As ***	0.16 ± 0.05	0.12 ± 0.04	0.18 ± 0.01	0.19 ± 0.01	0.12 ± 0.01	0.31 ± 0.01	0.17 ± 0.02	0.31 ± 0.02
Cd ***	0.030 ± 0.001	0.059 ± 0.001	0.057 ± 0.001	<LOD	0.013 ± 0.001	0.036 ± 0.001	0.015 ± 0.009	0.021 ± 0.002
Co ***	0.07 ± 0.01	0.02 ± 0.01	0.05 ± 0.01	0.05 ± 0.01	0.05 ± 0.01	0.06 ± 0.01	0.03 ± 0.01	0.05 ± 0.01
Cr ***	0.03 ± 0.01	0.06 ± 0.01	0.01 ± 0.01	<LOD	<LOD	0.08 ± 0.01	0.06 ± 0.01	<LOD
Mo ***	0.03 ± 0.01	0.03 ± 0.01	0.03 ± 0.01	0.04 ± 0.01	0.05 ± 0.01	0.05 ± 0.01	0.05 ± 0.01	0.08 ± 0.01
Ni ***	0.16 ± 0.01	0.18 ± 0.01	0.15 ± 0.01	0.08 ± 0.01	0.06 ± 0.01	0.12 ± 0.01	0.18 ± 0.01	0.03 ± 0.01
Pb ***	0.09 ± 0.01	0.07 ± 0.01	0.11 ± 0.01	0.03 ± 0.01	0.07 ± 0.01	0.11 ± 0.01	0.08 ± 0.01	0.15 ± 0.01
Se ***	0.30 ± 0.01	0.28 ± 0.01	0.11 ± 0.01	<LOD	0.11 ± 0.01	0.07 ± 0.01	0.12 ± 0.01	0.23 ± 0.01
V ***	0.024 ± 0.001	0.067 ± 0.001	0.018 ± 0.005	<LOD	<LOD	0.018 ± 0.001	0.061 ± 0.004	<LOD

A nonparametric multiple test (Kruskal–Wallis) was applied with *p*-values: ns not significant; *** 0.001. ‘±’ indicates SD of the measurements (*n* = 5). <LOD—concentration of the specific element lower than the limit of detection.

**Table 2:** Concentrations (mg/kg) of elements in blueberry samples from different countries.

Element	Peru	Chile	Uruguay	Argentina	Morocco	Spain	Germany	Poland	Latvia
K ***	6592 ± 59	7126 ± 66	5583 ± 72	6505 ± 26	7058 ± 94	5372 ± 63	4443 ± 31	6092 ± 62	4144 ± 280
Ca ***	1293 ± 32	520 ± 75	508 ± 40	1028 ± 68	834 ± 88	477 ± 30	550 ± 58	360 ± 16	371 ± 31
Mg ***	459 ± 34	423 ± 44	370 ± 40	438 ± 34	475 ± 40	388 ± 14	342 ± 20	275 ± 1	322 ± 14
Na ***	90 ± 12	38 ± 7	20 ± 5	41 ± 9	134 ± 8	25 ± 3	22 ± 2	45 ± 4	34 ± 4
P ***	586 ± 14	804 ± 14	767 ± 11	882 ± 27	918 ± 53	907 ± 24	533 ± 26	875 ± 50	746 ± 26
S ***	531 ± 46	655 ± 64	578 ± 32	620 ± 41	587 ± 50	658 ± 12	363 ± 17	576 ± 12	619 ± 24
Al ***	33.2 ± 6.8	14.9 ± 2.8	14.6 ± 1.9	33.4 ± 3.2	9.7 ± 8.1	5.9 ± 0.4	7.1 ± 0.3	10.7 ± 0.9	6.9 ± 0.4
B ***	3.77 ± 0.46	3.83 ± 0.73	1.82 ± 0.60	3.48 ± 0.34	3.92 ± 0.20	2.22 ± 0.11	2.79 ± 0.23	2.97 ± 0.11	2.62 ± 0.21
Ba ***	2.74 ± 0.08	2.82 ± 0.09	2.73 ± 0.81	8.54 ± 0.71	0.45 ± 0.08	0.54 ± 0.05	2.96 ± 0.43	0.68 ± 0.04	1.02 ± 0.18
Cu ***	1.90 ± 0.11	7.91 ± 0.33	3.15 ± 0.46	3.90 ± 0.39	2.83 ± 0.63	1.54 ± 0.16	2.03 ± 0.17	1.58 ± 0.08	2.69 ± 0.41
Fe ***	31.7 ± 4.1	13.3 ± 2.8	18.4 ± 2.3	26.0 ± 2.9	7.5 ± 3.8	14.9 ± 0.5	14.4 ± 1.4	16.0 ± 0.5	19.0 ± 1.8
Mn ***	27.5 ± 3.4	21.5 ± 4.9	21.9 ± 8.0	117.2 ± 6.9	18.3 ± 5.4	15.6 ± 1.1	7.2 ± 0.5	36.1 ± 1.8	13.2 ± 1.3
Si ***	88.3 ± 7.1	69.3 ± 6.9	85.6 ± 4.7	144.3 ± 11.4	81.8 ± 9.4	16.0 ± 2.4	27.5 ± 1.0	46.7 ± 2.8	34.8 ± 4.7
Sr ***	3.77 ± 0.34	1.76 ± 0.18	1.58 ± 0.40	3.70 ± 0.62	0.39 ± 0.08	0.36 ± 0.03	0.80 ± 0.06	0.31 ± 0.06	0.96 ± 0.09
Zn ***	3.5 ± 0.4	6.5 ± 1.3	4.0 ± 0.4	5.2 ± 0.1	6.1 ± 0.5	5.1 ± 0.1	3.7 ± 0.5	2.8 ± 0.1	3.97 ± 0.20
As ***	0.15 ± 0.01	0.03 ± 0.01	0.13 ± 0.02	0.14 ± 0.02	0.18 ± 0.06	0.01 ± 0.01	0.30 ± 0.03	0.04 ± 0.01	0.08 ± 0.01
Cd ***	0.030 ± 0.009	<LOD	0.016 ± 0.006	0.073 ± 0.006	0.012 ± 0.001	0.005 ± 0.001	0.030 ± 0.002	0.022 ± 0.006	0.02 ± 0.002
Co ***	0.03 ± 0.01	0.02 ± 0.01	0.10 ± 0.02	0.02 ± 0.001	0.08 ± 0.005	0.02 ± 0.001	0.01 ± 0.005	0.02 ± 0.008	0.02 ± 0.01
Cr ***	0.10 ± 0.008	0.29 ± 0.005	<LOD	0.06 ± 0.008	<LOD	0.01 ± 0.006	<LOD	0.04 ± 0.007	0.02 ± 0.001
Mo ***	0.50 ± 0.002	<LOD	<LOD	0.07 ± 0.02	0.20 ± 0.02	0.07 ± 0.02	0.03 ± 0.02	0.06 ± 0.02	0.04 ± 0.02
Ni ***	0.42 ± 0.015	0.60 ± 0.144	0.44 ± 0.065	0.26 ± 0.063	0.20 ± 0.05	0.04 ± 0.001	0.12 ± 0.037	0.22 ± 0.010	0.12 ± 0.03
Pb ***	0.12 ± 0.01	<LOD	0.11 ± 0.01	0.16 ± 0.031	0.13 ± 0.02	<LOD	0.13 ± 0.044	0.06 ± 0.01	0.05 ± 0.01
Se ***	0.24 ± 0.20	<LOD	0.37 ± 0.02	0.08 ± 0.01	0.30 ± 0.01	<LOD	0.08 ± 0.01	0.21 ± 0.01	0.13 ± 0.01
V ***	0.130 ± 0.02	<LOD	0.111 ± 0.01	0.025 ± 0.01	0.105 ± 0.01	0.04 ± 0.01	0.028 ± 0.01	0.03 ± 0.01	0.02 ± 0.01

A nonparametric multiple test (Kruskal–Wallis) was applied with *p*-values: ns not significant; *** 0.001. ‘±’ indicates SD of the measurements (*n* = 5). <LOD—concentration of the specific element lower than the limit of detection.

**Table 3:** Concentrations (mg/kg) of elements in bilberry samples from Northern Europe.

Element	Latvia	Lithuania	Finland	Norway
Ca ***	971 ± 32	1020 ± 46	1134 ± 72	1161 ± 56
K ***	5662 ± 66	5755 ± 116	5083 ± 72	5339 ± 54
Mg ***	436 ± 34	439 ± 43	471 ± 14	532 ± 28
Na ***	15 ± 4	9 ± 3	9 ± 3	42 ± 2
P ***	868 ± 14	1021 ± 12	1074 ± 18	1234 ± 15
S ***	791 ± 18	741 ± 26	617 ± 21	815 ± 21
Al ***	27.8 ± 6.8	20.7 ± 3,2	15.9 ± 7.1	16.8 ± 1.8
B ***	4.52 ± 0.4	4.84 ± 0.73	5.17 ± 0.53	6.28 ± 0.34
Ba ***	9.71 ± 0.08	10.16 ± 0.09	10.34 ± 0.07	12.73 ± 0.08
Cu ***	4.22 ± 0.39	5.35 ± 0.32	3.57 ± 0.29	3.42 ± 0.42
Fe ***	16.5 ± 2.3	81.8 ± 3.2	10.9 ± 2.8	18.1 ± 2.9
Mn ***	32.7 ± 1.1	243.3 ± 11.8	154.8 ± 21.2	216.6 ± 18.2
Si ***	12.7 ± 4.7	39.6 ± 5.9	26.7 ± 3.2	20.9 ± 3.9
Sr ***	1.14 ± 0.40	2.36 ± 0.62	2.71 ± 0.28	2.14 ± 0.48
Zn ***	6.5 ± 0.4	7.8 ± 0.4	5.5 ± 0.5	7.6 ± 0.3
As ***	0.12 ± 0.01	0.16 ± 0.01	0.03 ± 0.01	0.12 ± 0.02
Cd ***	0.049 ± 0.001	0.033 ± 0.002	0.021 ± 0.006	0.033 ± 0.004
Co ***	0.070 ± 0.01	0.067 ± 0.03	0.102 ± 0.03	0.049 ± 0.02
Cr ***	0.213 ± 0.008	0.177 ± 0.008	0.149 ± 0.007	0.297 ± 0.009
Mo ***	0.073 ± 0.002	0.091 ± 0.002	0.281 ± 0.002	0.096 ± 0.002
Ni ***	0.428 ± 0.019	0.455 ± 0.015	0.322 ± 0.013	0.089 ± 0.016
Pb ***	0.147 ± 0.012	0.456 ± 0.023	0.213 ± 0.016	0.247 ± 0.037
Se ***	0.427 ± 0.027	0.347 ± 0.022	0.646 ± 0.025	0.619 ± 0.023
V ***	0.067 ± 0.014	0.157 ± 0.021	0.050 ± 0.018	0.071 ± 0.014

A nonparametric multiple test (Kruskal–Wallis) was applied with p values: ns not significant; *** 0.001. ‘±’ indicates SD of the measurements (*n* = 9).
